# The role of self-compassion in diabetes management: A rapid review

**DOI:** 10.3389/fpsyg.2023.1123157

**Published:** 2023-03-30

**Authors:** Courtney Sandham, Elmari Deacon

**Affiliations:** ^1^Compres Research Unit, North-West University, Potchefstroom, South Africa; ^2^Optentia Research Unit, North-West University, Vanderbijlpark, South Africa

**Keywords:** self-compassion, diabetes, diabetes management, positive psychology, rapid review

## Abstract

**Aim:**

This study aimed to assemble and critically reflect on previously acquired insights from investigations that have already been conducted into the role of self-compassion in diabetes and its management.

**Methods:**

This study implemented a rapid review approach to assess the pre-existing knowledge in a time-sensitive manner. A rapid review involves the synthesis of existing knowledge using a simplified systematic review process.

**Results:**

A total of 16 articles were identified for this rapid review. The main findings from these articles included that self-compassion is associated with improved outcomes (psychologically and medically), self-compassion can be improved through interventions, and that many extraneous factors influence levels of self-compassion.

**Conclusion:**

It is apparent that self-compassion plays a rather significant role in the management of diabetes, and that interventions aimed at developing self-compassion showed success in improving health-related outcomes. It is suggested that future research should build on the possibility of using positive psychology interventions to improve the quality of life of those living with diabetes, and work to better understand the influence of aspects such as gender and diabetes duration on self-compassion.

## Introduction

Traditionally, the treatment of diabetes was only undertaken from a medical perspective, despite there being an increase in mental health issues among patients with diabetes (Hanmer, 2014; Ventura et al., 2019). Recently, there has been a shift in focus to the role of mental health in the effective management of chronic conditions, such as diabetes. This study will refer to the two major forms of diabetes: type 1 diabetes (T1D - insulin-dependent) and type 2 diabetes (T2D - non-insulin-dependent). T1D is diagnosed when the body does not produce enough insulin, whereas T2D refers to the body not using the produced insulin effectively (Roglic, 2016). Diabetes impacts the health of many of the body’s organs and often results in diabetes-associated complications such as loss of vision, decreased kidney functions, heart attacks, stroke, and limb amputations (Roglic, 2016; Karami et al., 2018).

The management of diabetes is no easy feat and requires many physical and psychological adaptations. However, effective self-management of this condition is vital as it prevents or delays the onset of diabetes-related health complications (Karami et al., 2018). Along with the potential physical effects, failure to achieve an optimal HbA1c level frequently increases psychological stress, which often leads to negative emotions, judgement from others (such as from doctors, family, and friends), and an overwhelming sense of failure and diabetes resentment (Ventura et al., 2019). For the previously stated reasons, a more Holistic approach must be adopted in the management and continued treatment of diabetes, including a dimension of mental health and well-being, such as self-compassion.

Mastering the art of being self-compassionate leads to many positive effects, both physiological and psychological. In short, self-compassion can be defined as the ability to show kindness and understanding towards oneself, particularly in times of hardship (Gilbert, 2018). Ventura et al. (2019) conducted a study in which they concluded that an increase in levels of self-compassion results in an overall decrease in anxiety, depression, and stress, while also leading to an increase in health-promoting behaviors. This may be attributed to the strengthening of a healthy relationship with oneself that inevitably follows when engaging in self-compassionate behaviors. Self-compassion is associated with other tenets of positive psychology, such as mindfulness, self-care, self-efficacy, and family empowerment (Whitebird et al., 2017; Rahmani et al., 2020; Loseby et al., 2022). These various facets need to be further explored to determine the role they could play in the development of self-compassion and, further, improved diabetes management.

However, it seems apparent that self-compassion is essential in reaching a state of prime physical and mental functioning. Hence, self-compassion may provide a way to improve the management and treatment of chronic conditions such as diabetes (Ferrari et al., 2017; Jackson, 2018; Tanenbaum et al., 2018; Morrison et al., 2019; Ventura et al., 2019; Rahmani et al., 2020; Akbari et al., 2022; Loseby et al., 2022).

Persons with diabetes frequently struggle with a variety of mental health issues as a result of the daily stresses that come with living with this chronic condition. Around 20% of people living with diabetes experience prominent levels of distress concerning their diabetes, while 12% appear to be living with major depression (Friis et al., 2016; Ventura et al., 2019). Along with symptoms of depression and distress, persons with diabetes have a four times higher chance of experiencing symptoms of anxiety; and have an increased risk of being victims of stereotyping, stigma, discrimination, and judgements made by others on their condition (Ferrari et al., 2017; Ventura et al., 2019).

With the factors indicated earlier in mind, there is no doubt that the psychological component of living with this condition must be considered when creating a suitable diabetes care plan (Charzyńska et al., 2020). Karami et al. (2018) argue that there is a great need for psychological interventions to help people accept their condition, equip them with the tools to engage in behavioral changes, and eliminate the psychological barrier between people and their effective control of the condition.

Self-compassion may provide the missing link to the effective management of this condition. This is evident in the studies that concluded that increased levels of self-compassion led to increased life satisfaction, more effective self-management behaviors, better glucose control, and a more optimal HbA1c (Karami et al., 2018; Charzyńska et al., 2020). Furthermore, studies in which interventions were conducted to improve levels of self-compassion found a statistically meaningful reduction in HbA1c levels, and an increase in general mental health, once the participants had completed the intervention training (Friis et al., 2016; Tanenbaum et al., 2020). Therefore, it is apparent that this relationship between self-compassion and diabetes management exists and needs further investigation. Tanenbaum et al. (2020) went so far as to coin the term “diabetes-specific self-compassion,” showing the undeniable connection between these two components. The information reviewed in this study will provide a basis for existing knowledge in this field and hopefully lay the groundwork for future studies.

## Methods

### Ethics statement

Ethical approval was granted for this study by the Health Research Ethics Committee (HREC) of the North-West University (NWU-00098-22-A1). Furthermore, no conflict of interest was declared by either reviewer, and both reviewers had undergone ethics training within the last 3 years. The primary reviewer ensured that ethics were upheld throughout the research process by being rigorous, responsible, and transparent with the data and engaging in continuous discussion and interaction with the secondary reviewer.

To ensure rigor was upheld throughout the study, four criteria were met: credibility, transferability, dependability, and confirmability (Lincoln et al., 1985). Credibility was ensured through reflexivity (keeping a reflective journal), peer examination (getting the article critically revised by experts), and structural coherence (integrating the data and comparing it with other literature). Dense descriptions of the method, data collection, and data analysis process allowed for the construct of transferability to be upheld. Dependability was ensured through the code-recoder procedure, using dense description, and keeping an audit trail. Lastly, confirmability was ensured through maintaining an audit trail, reflexivity, and triangulation, (which refers to the process of looking at the topic from different perspectives) (Lincoln et al., 1985).

### Review approach

A rapid review approach was implemented as the goal of this study was to assess what is already known on the topic of interest in a time-sensitive manner. A rapid review involves synthesizing existing knowledge through the implementation of a simplified systematic review process, enabling the reviewers to meet said goal (Grant and Booth, 2009; Dobbins, 2017). The five-step approach recommended by Dobbins (2017) was implemented to ensure that scientific rigor was upheld throughout the completion of this rapid review. The five steps are as follows:

#### The defining of a practice question

After a thorough literature review, the practice question was defined as: What conclusions may be drawn from available literature on the role of self-compassion in diabetes and its management?

#### Searching for research evidence

The search engines used to identify literature for this study included Google Scholar, LexisNexis, EBSCOhost, Emerald Insight Journals, JSTOR Journals, Juta, Sabinet Online, African Journals, ScienceDirect, Scopus, Web of Science, Boloka: NWU Institutional Repository (NWU-IR), and EBSCO Discovery Service (EDS). Keywords included in the search were ‘self-compassion,’ ‘diabetes,’ and ‘diabetes management,’ and they were combined in the following way: (self-compassion OR self compassion) AND (diabetes OR diabetes management OR diabetes control OR diabetes self-management). ‘Mindfulness’ was not included in the search terms as, although it is relevant to the topic, it encompasses a different field of study beyond the scope of this research. Once the search had been completed, the researcher found further literature by reviewing the resources used by the authors to expand the search further.

The inclusion criteria of literature for this review study were that the literature was published between the years 2014 to 2022 and was deemed scientifically sound. This time range was selected to include the latest literature and to ensure the information selected was relevant, given the acceleration of scientific research in recent years. The year 2014 was chosen as this was the year in which the first publication regarding self-compassion and diabetes management was made (Hanmer, 2014). The following literature was included as it was believed to have enough scientific rigor to allow for its inclusion: full-text journals, peer-reviewed studies, quantitative studies, qualitative studies, and mixed-method studies. Published PhD theses and master’s dissertations were also included as their studies would have gone through the necessary ethical considerations and would likely adhere to the principles of scientific rigor (de Klerk and Pretorius, 2019). In the search, no articles published in other languages relevant to the topic were found; therefore, none were included.

**Figure 1:**
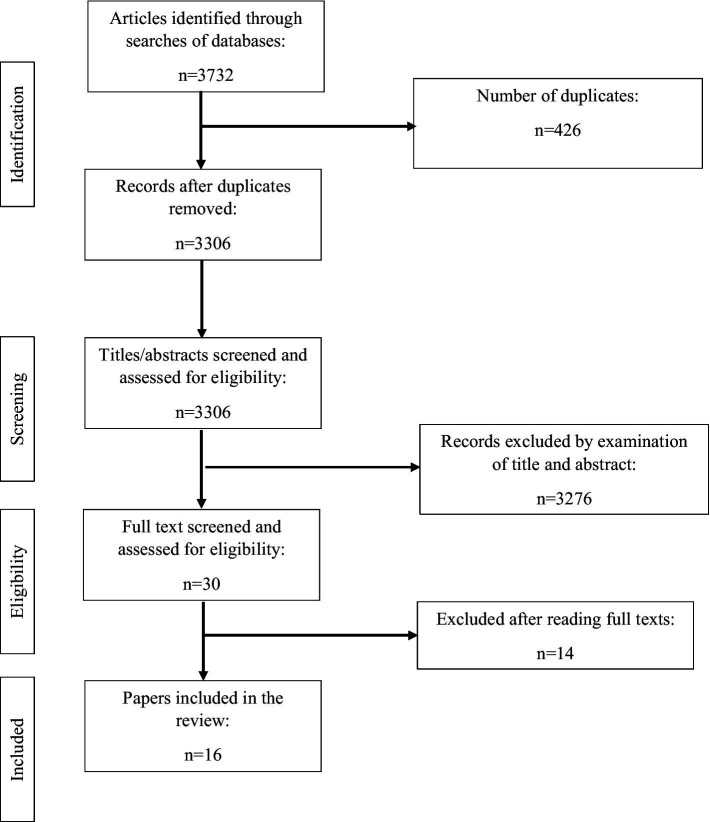
PRISMA flow diagram showing article selection.

The relevance of peer-reviewed literature in this rapid review was determined using the title and abstract. The process of eliminating articles was completed independently by each reviewer and consisted of them reading the gathered literature and deciding if it met the inclusion–exclusion criteria previously mentioned (as seen in Figure 1).

#### The critical appraisal of the literature gathered

The primary and secondary reviewers manually undertook the appraisal process. Once the initial list of literature to be included in the rapid review had been collected, the primary and secondary reviewers assessed the articles independently to determine if the findings were meaningful, reliable, valid, and relevant to the study (Dobbins, 2017; Wilson et al., 2021).

This analysis and assessment process was done by reading the articles and deciding if the research they report on was methodologically sound. The JBI’s (2022) critical appraisal tools were used to help the reviewers determine if the literature was appropriate for inclusion in this rapid review and met the ethical standards of this study. Depending on the type of literature under consideration, different checklists were used to assist the reviewers in assessing the trustworthiness, relevance, and results of the studies under consideration (JBI, 2022). The main checklists used included the JBI Critical Appraisal Checklist for Qualitative Research, the JBI Checklist for Quasi-Experimental Studies, and the JBI Checklist for Randomized Controlled Trials (JBI, 2022).

#### The synthesis of the information collected

After literature had been read in its entirety, relevant information based on the research question was extracted; including the author(s), the date and type of publication, in which country the publication was written, the aim of the publication, the research design, the outcomes measures in the study, the demographics of participants involved, the main findings of the publication, and the limitations identified by the authors (Dobbins, 2017). All data relating to the topic were included to prevent the omission of findings or results that may have been relevant to the synthesis process. The specific data components that were analyzed included details of self-compassion (what it is, why it is essential, and what interventions exist); details regarding the management of diabetes (what effective management involves and how to achieve it); findings or results (the role, if any, that self-compassion plays in diabetes management); and the conclusions (the researchers’ findings or results relating to the role of self-compassion in diabetes management, and the subsequent recommendations made on the implementation of interventions).

A narrative synthesis method was used during the search and compilation of the rapid review, as the goal of the proposed research was to synthesize results from various sources into a single document, namely this rapid review (Dobbins, 2017). A narrative synthesis allowed for a complete interpretation of the collected evidence (Garritty et al., 2021). The steps implemented to complete this narrative synthesis step were similar to the three steps presented by Dobbins (2017): the extraction of relevant information, the summation of results, and the formalization of conclusions.

A data extraction table (Table 1), as described by Dobbins (2017), was used to organize, and analyze the data. Using a data extraction table enabled the easy identification of the similarities and differences across the studies, thereby aiding the review of the literature (Dobbins, 2017). The reviewers remained cognizant that rapid reviews run the risk of bias and ensured to the best of their abilities that no relevant information was omitted by continuously consulting with one another (Grant and Booth, 2009).

**Table 1:** Data extraction table.

Article	Country	Aim	Design	Measures	Participants	Main findings	Limitations
Akbari, M., Seydavi, M., Rowhani, N. S., & Nouri, N. (2022). Psychological predictors of treatment adherence among patients with diabetes (types I and II): Modified information-motivation-behavioural skills model. *Clinical Psychology & Psychotherapy*, 1–13.	Iran	To determine whether there is a difference in the pattern of adherence between patients with types I and type II diabetes (T1D and T2D, respectively). To compare patients with diabetes to determine how they differ, or are similar, in terms of treatment adherence, interpersonal communication between staff and patients, perceived social support, distress intolerance, self-compassion and illness perception.	A cross-sectional, observational study.	Treatment adherence (as measured by the Treatment Adherence for Chronic Disease Questionnaire), interpersonal communication between staff and patients (as measured by the Interpersonal Communication Scale), illness perception (as measured by the Brief Illness Perception Questionnaire), perceived social support (as measured by the Multidimensional Scale of Perceived Social Support), distress intolerance (as measured by the Distress Tolerance Scale), and self-compassion (as measured by the short form of the Self-Compassion Scale).	1,125 participants (475 = T1D, 472 = T2D, 178 = diabetes due to other condition), 33.3 = mean age, 55.02% = women, 55.73% = single, ethnicity not reported, 6.01 years = diabetes duration, HbA1c not reported, and 40.88% = hold a diploma.	Self-compassion and interpersonal communication between staff and patients were significant predictors of treatment adherence among patients with T1D, T2D and diabetes due to other medical conditions. Distress intolerance was a significant predictor of treatment adherence only in patients with T2D. Above and beyond demographic features, self-compassion, interpersonal communication between patients and health care staff, distress intolerance, perceived social support and illness perception were significant predictors of treatment adherence among patients with diabetes.	The cross-sectional and observational nature of findings would not allow for conclusions on causality, temper and findings temporality. Reliance on self-report measurement and lack of a longitudinal follow-up to assess change over time. No data was collected on pharmacological treatments that may have acted as confounding variables. Participants’ psychological status may have been affected by the COVID-19 stressor, influencing treatment adherence.
Charzynska, E., Kocur, D., Dzialach, S., & Brenner, R. E. (2020). Testing the indirect effect of type 1 diabetes on life satisfaction through self-compassion and self-coldness. *Mindfulness, 11*(11), 2,486–2,493.	Poland	To explore the relationship between duration of diabetes, positive and negative components of self-compassion, and life satisfaction.	A cross-sectional, web-based study.	Self-compassion [as measured by the Self-Compassion Scale (SCS)], satisfaction with life [as measured by the Satisfaction with Life Scale (SWLS)], and sociodemographic and diabetes-related measures (as measured by self-report data).	112 participants (112 = T1D, 0 = T2D), 28.29 = mean age, 80.4% = women, 66.67% = intimate relationship, ethnicity not reported, 13.75 years = diabetes duration, HbA1c average not reported, and education level not reported.	Diabetes duration was related to lower self-coldness, but not to self-compassion. Both self-coldness and self-compassion are strongly correlated with life satisfaction. Diabetes duration had a significant indirect effect on life satisfaction through self-coldness, not self-compassion.	Small sample size, limit in generalizability as sample were mostly women, did not control for comorbidity in analysis, design used does not allow for predictions in directions of relationship between variables, use of non-specific measure of self-compassion and not a diabetes-specific one.
Ferrari, M., Cin, M. D., & Steele, M. (2017). Self-compassion is associated with optimum self-care behavior, medical outcomes and psychological well-being in a cross-sectional sample of adults with diabetes. *Diabetic Medicine, 34*(11), 1,546–1,553.	Australia	To investigate the role of self-compassion in diabetes outcomes.	A cross-sectional study.	Demographic information (as measured by self-reported data), medical information (as measured by self-reported HbA1C, duration of diagnosis, type of diagnosis, and most recent reading), self-compassion [as measured by the Self-Compassion Scale-Short Form (SCS-SF)], self-management behavior (as measured by the Diabetes Self-Management Questionnaire) and psychological well-being (as measured by the Well-Being Questionnaire).	310 participants (203 = T1D, 73 = T2D, 28 = Gestational diabetes, 6 = other), 37.6 = mean age, 81.3% = women, 42.9% = married, ethnicity not reported, diabetes duration not reported, 7.7% = HbA1c, and education level not reported.	Self-compassion had the most consistent association with better outcomes, including all forms of self-management behavior, HbA1c levels, and psychological well-being. Internal locus of control was also significantly associated with better well-being and HbA1c outcomes. External locus of control and social support were associated with poorer outcomes.	Challenges to validity of findings as it was an online questionnaire, self-report relies on honesty and diligence of participants, HbA1c was also self-reported or unreported, other relevant psychosocial predictors may have not been included.
Friis, A. M., Johnson, M. H., Cutfield, R. G., & Consedine, N.S. (2015). Does kindness matter? Self-compassion buffers the negative impact of diabetes-distress on HbA1c. *Diabetes Spectrum, 28*(4), 252–257.	New Zealand	To assess the specific operationalization of negative emotionality that best predicted HbA1c, and to test whether self-compassion would buffer HbA1c in patients with diabetes against the negative effects of distress.	A cross-sectional study.	Symptoms of major depressive disorder (as measured by the PHQ-19), diabetes-specific distress [as measured by the Diabetes Distress Scale 2 (DDS-2)], and self-compassion [as measured by the Self-Compassion Scale (SCS)].	110 participants (67 = T1D, 43 = T2D), 47.6 = mean age, 65.45% = women, relationship status not reported, 73.6% = New Zeeland European, 16.7 years = diabetes duration, 8.5% = HbA1c, and education level not reported.	Diabetes-specific distress was a better predictor of HbA1c than depression. Self-compassion moderated the relationship between distress and HbA1c (higher distress predicted higher HbA1c at lower levels of self-compassion).	Self-report measure, participants were self-selected, use of short form measure of DDS-2, directionality cannot be inferred due to research design (cross-sectional), limited ethnic diversity within sample, comorbidities or diabetes complications were not assessed.
Friis, A. M., Johnson, M. H., Cutfield, R. G., & Consedine, N.S. Does kindness matters: A randomized controlled trial of a mindful self-compassion intervention improves depression, distress, and HbA1c among patients with diabetes. *Diabetes Care, 39*(11), 1963–1971.	New Zealand	To evaluate the effects of self-compassion training on mood and metabolic outcomes among patients with diabetes.	A randomized controlled trial study with an eight-week mindful self-compassion intervention program. There was a waitlist control group, and measurements were taken at baseline, at eight-week/postvention, and at a 3 month follow up.	Self-compassion [as measured by the Self-Compassion Scale (SCS)], symptoms of major depressive disorder [as measured by the 9-Item Patient Health Questionnaire (PHQ-9)], diabetes-specific distress [as measured by the 17-Item Diabetes Distress Scale (DDS)], and glycemic control (as measured by HbA1c).	63 participants (46 = T1D, 17 = T2D), 44.37 = mean age, 68.25% = women, relationship status not reported, 73.02% = New Zealand European, 16.84 years = diabetes duration, 8.94% = HbA1c, and education level not reported.	MSC training increased self-compassion and produced statistically and clinically significant reductions in depression and diabetes distress. These results were maintained at the three-month follow-up. Participants in the intervention group also averaged a clinically and statistically meaningful decrease in HbA1c between baseline and follow-up. No changes were present in wait-list control group.	Findings are generalizable only to those who volunteered for the RCT, more than one half of the sample presented with mood problems, absence of an active control group.
Jackson, K. (2018). *Exploring the role of self-compassion in adolescent wellbeing and type 1 diabetes management*. [Doctoral dissertation]. University of East Anglia.	UK	To examine the association between self-compassion and subjective wellbeing, and to investigate self-compassion as a correlate of effective disease management in adolescents with type 1 diabetes, as indicated by measures of glycemic control and regimen adherence	A quantitative, cross-sectional study.	Glycemic control (as measured by HbA1c), diabetes regimen adherence (as measured by the 14-Item Self-Care Inventory), self-compassion [as measured by the Self-Compassion Scale (SCS)], emotional distress (as measured by the Paediatric Index of Emotional Distress [PI-ED)], critical/intrusive parental diabetes behavior [as measured by the Diabetes Family Behavior Checklist (DFBC)], demographics and diabetes information (as measured by self-reported data).	52 participants (52 = T1D, 0 = T2D), 14.87 = mean age, 44.2% = women, relationship status not reported, ethnicity not reported, 7.06 years = diabetes duration, 8.2% = HbA1c, and education level not reported.	Self-compassion was found to predict improved glycaemic control and regimen adherence, outcomes linked to a reduced risk of short- and long-term health complications. Impaired self-soothing was also discovered to mediate the relationship between emotional distress and poorer diabetes regimen adherence.	Scarcity of evidence relevant to research question, shortage of experimental evidence (review was limited to cross-sectional), other moderators need to be accounted for, more cross-cultural research is required.
Kane, N. S., Hoogendoorn, C. K., Tanenbaum, M. L., & Gonzalez, J. S. (2018). Physical symptom complaints, cognitive emotion regulation strategies, self-compassion and diabetes distress among adults living with Type 2 diabetes. *Diabetic Medicine 35*(12), 1,671–1,677.	USA	To examine illness burden, and positive and negative ways of thinking and relating to oneself at times of stress, as independent correlated of diabetes distress, cross-sectionally and longitudinally.	A cross-sectional and longitudinal study.	Physical symptom burden [as measured by the Illness Perception Scale-Revised (IPQ-R)], cognitive emotion regulation strategies [as measured by the 36-Item Cognitive Emotion Regulation Questionnaire (CERQ)], and self-compassion [as measured by the 26-Item Self-Compassion Scale (SCS)].	120 participants (0 = T1D, 120 = T2D), mean age not reported, 64.2% = female, relationship status not reported, 61.7% = black, 12.9 years = diabetes duration, 8.0% = HbA1c, and 32.5% = some college experience.	Baseline diabetes distress was associated with greater use of negative cognitive emotion regulation strategies, a greater tendency towards self-criticism, self-judgement and over-identification, and a greater physical symptom burden. Baseline physical symptoms and negative cognitive emotion regulation were independently associated with baseline diabetes distress. Baseline physical symptoms and negative aspects of self-compassion significantly predicted diabetes distress over 3 months. Positive aspects of cognitive emotion regulation and self-compassion were not independently associated with diabetes distress cross-sectionally or longitudinally.	A cross-sectional design limits the ability to make causal inferences; two time points over 3 months were not sufficient to examine change over time; there is a degree of construct and measurement overlap between cognitive emotional regulation and self-compassion which may have limited the ability to identify independent roles for these factors in relation to diabetes distress; did not differentiate among types of physical symptoms and those attributed to diabetes or other causes; small sample size.
Karami, J., Rezaei, M., Karimi, P., & Rafiee, Z. (2018). Effectiveness of self-compassion intervention training on glycemic control in patients with diabetes. *Journal Kermanshah University Medical Sciences, 22*(2).	Iran	To investigate the effectiveness of self-compassion training on glycemic control in patients with type II diabetes.	A quasi-experimental study with an eight-session self-compassion training, with a pretest-posttest design and a control group.	Demographic details (as measured by self-reported information) and blood glucose level (as measured by a self-reported blood glucose reading).	20 participants (0 = T1D, 20 = T2D), 43.98 = mean age, gender not reported, relationship status not reported, ethnicity not reported, diabetes duration not reported, HbA1c not reported and education level not reported.	After the intervention, the mean score of the experimental group were significantly lower than that of the control group. Self-compassion training is effective in glycemic control in patients with diabetes.	None are mentioned in the article.
Kilic, A., Hudson, J., Scott, W., McCracken, L. M., & Hughes, L. D. (2022). A 12-month longitudinal study examining the shared and unique contributions of self-compassion and psychological inflexibility to distress and quality of life in people with Type 2 Diabetes. *Journal of Psychosomatic Research, 155.*	UK	To examine the shared and unique utility of self-compassion and psychological flexibility in predicting distress and quality of life (QoL) outcomes over time.	An online longitudinal study with measures taken at baseline; and six and 12 months follow-ups.	Demographic information and health status (as measured by self-report questionnaire), depressive symptoms [as measured by Whooley Questions and the Patient Health Questionnaire (PHQ-8)], anxiety symptoms [as measured by the Generalized Anxiety Disorder-7 (GAD-7)], diabetes distress [as measured by the Problem Areas in Diabetes (PAID)], quality of life (as measured by the EQ-5D-3 l Health Questionnaire Visual Analogue Scale), self-compassion [as measured by the Self-Compassion Scale (SCS)], and psychological inflexibility [as measured by the Acceptance and Action Questionnaire-2 (AAQ-2)].	173 participants (0 = T1D, 173 = T2D), 58.3 = mean age, 60.1% = women, 57.9% = living with someone, 92.5% = white, 10.18 years = diabetes duration, HbA1c not reported, and education level not reported.	Significant negative correlations between self-compassion and psychological inflexibility. Both had significantly large correlations with distress, but not QoL over time. Psychological inflexibility predicted depression, anxiety, and QoL; while self-compassion did not uniquely predict any of the outcomes.	Low completion rates and small sample size; data collection was completed during the COVID-19 pandemic and so participants may have been experiencing higher levels of disress and lower QoL due to this; self-reporting bias; only a limited number of variables were controlled for; findings may not generalize.
Loseby, P., Schache, K., Cavadino, A., Young, S., Hofman, P. L., & Serlachius, A. (2021). The role of protective psychological factors, self-care behaviours, and HbA1c in young adults with type 1 diabetes. *Behavioral Aspects of Diabetes, 23*(3), 380–389.	New Zealand	To investigate whether protective psychological factors in young adults with type 1 diabetes are associated with more optimal self-care behaviors and HbA1c, and to explore possible mediators between protective psychological factors and HbA1c.	A cross-sectional study.	Demographic information and HbA1c (as measured by the National Health Index), optimism [as measured by the revised version of the Life Orientation Test (LOT-R)], anxiety and depression [as measured by the Hospital Anxiety and Depression Scale (HADS)], self-compassion [as measured by the Self-Compassion Scale-Short Form (SCS-SF)], positive efficacy expectancies (as measured by the Generalized Self-Efficacy Scale), stress [as measured by the 10-item Perceived Stress Scale (PSS-10)], and self-care behaviors [as measured by the Self-Care Inventory-Revised Version (SCI-R)].	113 participants (113 = T1D, 0 = T2D), 20 = mean age, 53.1% = women, marital status not reported, 65% = New Zealand European,10.68 years = diabetes duration, 9.1% = HbA1c, and education level not reported.	Higher positive efficacy expectancies were associated with more optimal HbA1c and more optimal self-care behaviours. Higher levels of self-compassion were associated with more optimal self-care behaviors. Self-care behaviors mediated the relationship between all the protective psychological factors and more optimal HbA1c, and lower stress also mediated relationship between higher self-compassion and more optimal HbA1c.	No inclusion of measures of socioeconomic status which is known to be strongly associated with diabetes outcomes; cannot assume causality due to cross-sectional design.
Morrison, A. E., Zaccardi, F., Chatterjee, S., Brady, E., Doherty, Y., Robertson, N., Hadjiconstantinou, M., Daniels, L., Hall, A., Khunti, K., & Davies, M. J. (2019). Self-compassion, metabolic control and health status in individuals with type 2 diabetes: A UK observational study. *Experimental and Clinical Endocrinology & Diabetes, 129*(06), 413–419.	UK	To explore levels of self-compassion in individuals with type 2 diabetes (T2DM) and their association with levels of depression, diabetes-related distress and glycaemic control.	A cross-sectional study.	Demographic details and medical information/history (as measured by self-reported data and blood results), self-compassion [as measured by the Self Compassion Scale (SCS)], depression symptoms [as measured by the Patient Health Questionnaire (PHQ-9)], and diabetes-specific-distress [as measured by the Diabetes Distress Scale (DDS-17)].	176 participants (0 = T1D, 176 = T2D), 66 = mean age, 31.8% = female, relationship status not reported, 83% = white, 11 years = diabetes duration, 7.3%,=HbA1c and education level was not reported.	Higher levels of self-compassion and lower levels of depressive symptoms were associated with significantly better long-term diabetes control.	Small sample size of only patients with T2DM.
Rahmani, S., Mansoobifar, M., Seirafi, M., Ashayeri, H., & Bermas, H. (2020). Effectiveness of family empowerment therapy based on self-compassion on self-care and glycosylated hemoglobin in female patients with type 2 diabetes mellitus: A randomized controlled clinical trial. *Women’s Health Bulletin, 7*(2), 33–42.	Iran	To determine the effectiveness of family empowerment therapy based on self-compassion on self-care and glycosylated hemoglobin in female patients with type 2 diabetes mellitus.	A randomized controlled clinical trial with a control group, pre-test, post-test and follow-up measurements. A family empowerment therapy based on self-compassion was used as an 8 weeks intervention program.	Self-care [as measured by the Summary of Diabetes Self-Care Activities (SDSCA)], and glycosylated hemoglobin (as measured by HbA1c).	60 participants (0 = T1D, 60 = T2D), mean age not reported, 100% = women, 57.7% = married, ethnicity not reported, diabetes duration not reported, 6.79% = HbA1c, and 57.7% = Bachelor’s degree.	Significant difference after the intervention between the experimental and control groups regarding self-care and HbA1c. Comparison of means indicates the effectiveness of treatment in improving self-care and reducing HbA1c.	Small sample size; use of self-reported instruments; sample limited to female patients.
Ringdahl, B. A. (2019). *Man your meter: The mediating roles of self-compassion and self-efficacy between gender role conflict and diabetes self-care, diabetes distress, and glucose control in men with diabetes.* [Doctoral dissertation]. University of St. Thomas.	USA	To examine self-efficacy and self-compassion as mediators that further explain how men’s levels of gender role conflict may subsequently influence diabetes-related health outcome variables.	A quantitative, cross-sectional, survey study.	Demographic information (as measured by a self-report questionnaire), gender role conflict [as measured by the 37-Item Gender Role Conflict Scale (GRCS)], depression [as measured by the 21-Item Beck Depression Inventory-II (BDI-II)], self-compassion [as measured by the Self-Compassion Scale (SCS)], self-efficacy [as measured by the 10-Item self-administered General Self-Efficacy Scale (GSES)], diabetes distress [as measured by the Diabetes Distress Scale-17 (DDS-17)], diabetes self-management [as measured by the 16-Item Diabetes Self-Management Questionnaire (DSMQ)], glycemic management (as measured by HbA1c), and covariates (as measured by self-reported information).	146 participants (21 = T1D, 125 = T2D), 54.69 = mean age, 100% = men, 59.3% = married, 65.8% = white, 12.40 years = diabetes duration, 7.63% = HbA1C, and 28.1% = some college credit but no degree.	Multiple regression analyses found that gender role conflict correlated with measures of diabetes self-care and diabetes distress and that self-compassion mediated the relationship between gender role conflict and diabetes-related health outcomes.	Due to design of the study, the development and maintenance of GRC in the context of the lives of the participants cannot be fully understood; GRCS measures a limited number of behavioral domains; data was not randomized; self-reported measures; sample size was small.
Tanenbaum, M. L., Adams, R. N., Gonzalez, J. S., Hanes, S. J., & Hood, K.K. (2018). Adapting and validating a measure of diabetes-specific self-compassion. *Journal of Diabetes and Its Complications, 32*(2), 196–202.	USA	To adapt the Self-Compassion Scale and validate it for a diabetes-specific population.	A cross-sectional study.	Diabetes and demographic characteristics (as reported by self-report), convergent validity, diabetes empowerment [as measured by the 8-Item Diabetes Empowerment Scale-Short Form (DES-SF)], diabetes distress [as measured by the 28-Item Diabetes Distress Scale for Adults with T1D (DDS-T1)], glycemic control (as measured by HbA1c values), and discriminant validity: diabetes numeracy (as measured by the Diabetes Numeracy Test (DNT-5) which refers to the ability to interpret diabetes-related numbers and use these numbers to guide diabetes management tasks).	542 participants (542 = T1D, 0 = T2D), 41.4 = mean age, 65% = women, marital status not reported, 96.8% = white, 23.3 years = diabetes duration, 7.3% = HbA1c, and education level not reported.	Higher SCS-D was associated with less distress, greater empowerment, and lower HbA1c, and was not associated with numeracy.	The sample may not fully represent the larger population of adults with T1D; HbA1c data was only available for one-third of the population; the relationship may not be generalizable; the sample had high rates of device use.
Ventura, A. D., Nefs, G., Browne, J. L., Friis, A. M., Pouwer, F., & Speight, J. (2018). Is self-compassion related to behavioural, clinical, and emotional outcomes in adults with diabetes? Results from the second diabetes MILES - Australia (MILES-2) study. *Mindfulness, 10*(7), 1,222–1,231.	Australia	To determine the associations between self-compassion and diabetes-related health behaviors and clinical outcomes, and emotional health outcomes.	A cross-sectional study.	Self-compassion [as measured by the Self-Compassion Scale-Short Form (SCS-SF)], diabetes self-management behaviors [as measured by the Summary of Diabetes Self-Care Activities Questionnaire (SDSCA)], emotional outcomes [as measured by the 20-Item Problem Areas in Diabetes scale (PAID)], demographic and clinical characteristics (as measured by self-reported information, including HbA1c).	1907 participants (889 = T1D, 1,018 = T2D), 53.01 = mean age, 50% = women, relationship status not reported, ethnicity not reported, 14.90 years = diabetes duration, 7.3% = HbA1c, and education level not reported.	Self-compassion was significantly lower among those with severe diabetes distress or moderate-to-severe symptoms of depression and anxiety. Self-compassion was significantly associated with all specified outcomes, with the strongest association observed among the emotional outcomes. Self-compassion was found to be meaningfully associated with more optimal behavioral, clinical, and emotional outcomes in adults with diabetes.	Use of self-reported data; use of short-form of the self-compassion scale as opposed to the full version; use of a measure that was validated and adapted in the USA.
Whitebird, R. R., Kreitzer, M. J., Vazquez-Benitez, G., & Enstad, C. J. (2017). Reducing diabetes distress and improving self-management with mindfulness. *Social Work in Health Care, 57*(1), 28–65.	USA	To determine whether mindfulness-based stress reduction could reduce diabetes distress and improve management.	A one-arm pilot study in which an 8 week mindfulness-based stress-reduction (MSSBR) program intervention was implemented. Measures were taken at baseline and post-intervention.	Demographics (as measured by self-reported data), stress [as measured by the Perceived Stress Scale (PSS)], coping [as measured by the short-form version of the Coping Strategies Inventory (CSI-SF)], mental health [as measured by the Short-Form-12 Health Survey (SF-12)], social support [as measured by the Medical Outcomes Study Social Support Survey (MOS)], self-compassion [as measured by the short-form of the Self-Compassion Scale (SCS-SF)], diabetes-related distress [as measured by the Problem Areas in Diabetes Questionnaire (PAID)], self-management and self-efficacy [as measured by the Diabetes Empowerment Scale (DES)],and HbA1c (as measured by medical records).	31 participants (0 = T1D, 31 = T2D), 56.6 = mean age, 67.4% = women, 62.3% = married, 71% = white, diabetes duration not reported, 9.2% = HbA1c, and 38.7% = some post-secondary education.	Participants showed significant improvement in diabetes-related distress, psychological self-efficacy, and glucose control. Significant improvements in depression, anxiety, stress, coping, self-compassion and social support were also found.	As a single-arm pilot study, the design precludes causal inference, and only provides preliminary data to address the question of the effectiveness of MBSR. Small sample size and predominantly female with limited representation of minorities.

#### The identification of applicability and transferability issues

The last step, as identified by Dobbins (2017), was the identification of applicability and transferability issues. Burchett et al. (2013) defined applicability as the ability to implement an intervention in a new setting; while transferability refers to the process of determining if the results or findings of the study would be effective in a different setting than that of the original study. These concepts will be addressed in the results and discussion sections that follow.

## Results

In total, 16 publications were included in the final review: four from the USA, three from New Zealand, three from Iran, three from the UK, two from Australia, and one from Poland. Of the publications included, 11 used a cross-sectional design, three used a quasi-experimental research design, and two employed a randomized controlled trial. Three major themes were identified: self-compassion is associated with improved outcomes, self-compassion can be improved through interventions, and there are other factors which influence self-compassion, specifically gender and diabetes duration.

### Self-compassion is associated with improved outcomes

In eight of 16 studies, self-compassion was shown to be related to an improvement in regimen adherence and HbA1C levels and an increase in various psychological well-being domains (Ferrari et al., 2017; Jackson, 2018; Tanenbaum et al., 2018; Morrison et al., 2019; Ventura et al., 2019; Rahmani et al., 2020; Akbari et al., 2022; Loseby et al., 2022). One article (Kılıç et al., 2022) did not support this and found that self-compassion did not uniquely predict variables such as depression, anxiety, and quality of life. However, this article identified that a limitation of their study was that data collection was completed during the COVID-19 pandemic, in which participants may have been experiencing increased levels of distress, anxiety, and lower quality of life, due to extraneous variables beyond that of their diabetes (Kılıç et al., 2022).

Compelling evidence from this review indicates that self-compassion is meaningfully associated with more optimal behavioral, clinical, and emotional outcomes in individuals with diabetes (Ferrari et al., 2017; Morrison et al., 2019; Ventura et al., 2019; Akbari et al., 2022). Ferrari et al. (2017) identified that self-compassion was strongly correlated with an increased sense of psychological well-being and improved HbA1c levels. Self-compassion was also found to be meaningfully associated with more optimal self-care behaviors (Loseby et al., 2022). It is vital to understand this relationship, as improved self-care behaviors have been shown to improve regimen adherence and self-management behaviors which, in turn, reduce HbA1c levels (Ferrari et al., 2017; Jackson, 2018; Rahmani et al., 2020; Loseby et al., 2022).

Many psychological variables were identified in the articles that influence the relationship between self-compassion, improved HbA1c, and psychological well-being. These psychological variables included psychological inflexibility (Kılıç et al., 2022), interpersonal communication (Akbari et al., 2022), and diabetes-related distress and depression (Friis et al., 2015; Whitebird et al., 2017; Kane et al., 2018; Kılıç et al., 2022). Kılıç et al. (2022) identified a negative correlation between self-compassion and psychological inflexibility, meaning that when individuals displayed an increased level of self-compassion, they exhibited less psychological inflexibility or were more psychologically flexible. This is valuable information, as psychological inflexibility was also shown to predict other psychological variables such as depression, anxiety, and an individual’s sense of the quality of their life (Kılıç et al., 2022). Furthermore, Akbari et al. (2022) identified how interpersonal communication between staff and patients, self-compassion, and distress intolerance all influenced their treatment adherence.

Another confounding relationship identified in five articles was between self-compassion, diabetes distress/depression and HbA1c (Friis et al., 2015; Whitebird et al., 2017; Tanenbaum et al., 2018; Morrison et al., 2019; Ventura et al., 2019). Self-compassion was found to mediate the relationship between diabetes distress/depression and HbA1c in that higher levels of self-compassion were related to less distress/depression and lower HbA1c readings (Friis et al., 2015; Tanenbaum et al., 2020). This was reported to be significant as diabetes-specific distress/depression was a better predictor of HbA1c levels than general psychological depression (Friis et al., 2015).

### Self-compassion can be improved through interventions

Four studies included in this review reported on interventions in the form of self-compassion training (Friis et al., 2016; Whitebird et al., 2017; Karami et al., 2018; Rahmani et al., 2020). All the studies found a meaningful increase in glycemic control and a reduction in HbA1c levels. Friis et al. (2016) conducted an eight-week mindful self-compassion (MSC) intervention program in which the standard MSC protocol was strictly adhered to. MSC interventions aim to develop the cognitive, behavioral, and physical capacities to soothe and comfort oneself when distressed, using formal meditation and self-compassion practices (Friis et al., 2016). After conducting the interventions, the authors reported having found an increased level of self-compassion and a clinically significant reduction in depression, diabetes-related distress, and HbA1c levels (Friis et al., 2016). The authors confirmed these results using ANOVA testing.

In an eight-week mindfulness-based stress reduction (MBSR) program conducted by Whitebird et al. (2017), significant improvements in diabetes-related distress, glucose control, self-compassion, and other positive psychological characteristics were found. According to the authors, these findings suggest that MBSR may be an effective method for assisting individuals living with diabetes in better managing their diabetes and overall mental health.

An intervention by Karami et al. (2018) offered eight sessions of group-based self-compassion training to an experimental group, while the control group did not receive any training. Post-intervention, the mean blood glucose levels of the experimental group were reported to be significantly lower than that of the control group (with *p* < 0.001) (Karami et al., 2018). Therefore, it was concluded that self-compassion training is an effective way of increasing glycemic control in a group of people with diabetes (Karami et al., 2018).

On a more holistic level, Rahmani et al. (2020) conducted an eight-week family empowerment therapy intervention, based on self-compassion, among an experimental group. The control group continued to receive their usual hospital treatments during the therapy sessions. After conducting an analysis of variance, the authors found a significant difference between the experimental and control groups regarding their self-care and HbA1c levels post-intervention; further illustrating the effectiveness of self-compassion-based training on diabetes-related health outcomes (Rahmani et al., 2020).

### Other factors influencing self-compassion

Gender and diabetes duration were identified as factors that should be considered when investigating the relationship between self-compassion and diabetes management.

#### Gender

Of the studies in this review, eight included a sample of more than 65% women (Friis et al., 2015, 2016; Ferrari et al., 2017; Whitebird et al., 2017; Tanenbaum et al., 2018; Ringdahl, 2019; Charzyńska et al., 2020; Rahmani et al., 2020; Tanenbaum et al., 2020). Given that previous research has indicated that women tend to show lower levels of self-compassion than their male counterparts, the results of the studies should be interpreted with caution as they may have been influenced purely by the composition of the sample (Yarnell et al., 2018; Ferrari et al., 2022).

Ringdahl (2019) recognized the influence that gender could have on self-compassion and conducted an all-male study investigating the relationship between gender role conflict, self-care behaviors, and diabetes distress. This study concluded that self-compassion mediates the effects of gender role conflict on diabetes-related health outcomes (Ringdahl, 2019).

#### Diabetes duration

Diabetes duration was not reported in all the studies included in this review; however, of the ones that did report on it, 10 of them had a sample consisting of people that had been living with diabetes for a period of 10 years or longer (Friis et al., 2015, 2016; Kane et al., 2018; Tanenbaum et al., 2018; Morrison et al., 2019; Ringdahl, 2019; Ventura et al., 2019; Charzyńska et al., 2020; Kılıç et al., 2022; Loseby et al., 2022).

This is of note as Charzyńska et al. (2020) reported on the effects that diabetes duration has on self-coldness (a negative aspect of self-compassion). It was concluded that the longer an individual had diabetes (increased diabetes duration), the lower their levels of self-coldness (Charzyńska et al., 2020). However, they also found that diabetes duration had no impact on an individual’s levels of self-compassion.

## Discussion

This rapid review’s main objective was to determine self-compassion’s role in diabetes and its management. From the publications reviewed in this study, it can be concluded that although many psychological variables influence the role that self-compassion plays in the management of diabetes, it may provide a potential avenue through which psychological well-being could improve, regimen adherence could be increased, and HbA1c could be lowered.

Diabetes remains one of the major chronic conditions globally, affecting at least 34 million people in the United States alone (NCCDPHP, 2021). Effective diabetes management involves a healthy eating plan, engaging daily in some sort of physical activity, medication adherence, and the close management of blood glucose levels; all of which are emotionally, physically, and cognitively demanding activities (Roglic, 2016; Boggiss et al., 2020).

Given that a fundamental component of diabetes management is being able to learn from mishaps and being able to forgive oneself when failing to meet all aspects of daily management; it is clear that a concept such as self-compassion, in which treating oneself with kindness and concern while enduring negative events or experiences, may provide an important psychological cushion (Allen and Leary, 2010). This was made evident in the literature that concluded that self-compassion could improve levels of diabetes-related distress/depression, interpersonal communication, and psychological inflexibility (Friis et al., 2015, 2016; Whitebird et al., 2017; Kane et al., 2018; Morrison et al., 2019; Ventura et al., 2019; Tanenbaum et al., 2020; Akbari et al., 2022; Kılıç et al., 2022).

Diabetes-related distress/depression is a broad term used to describe the distress, negative mood, and emotional burden associated with managing diabetes daily (Friis et al., 2015; Kane et al., 2018). It is important to investigate this variable as when an individual with diabetes is experiencing this distress/depression, they are more likely to engage in self-judgement and self-criticism when they experience poor glucose control, which often leads to their taking fewer actions to manage their diabetes, further exacerbating the symptoms of diabetes-related distress/depression (Friis et al., 2015; Whitebird et al., 2017; Kane et al., 2018). A promising finding in the literature was identifying self-compassion as a potential resource that can be used to break this downward spiral. The possibility of self-compassion being a mediating factor in this relationship between diabetes-specific distress/depression, glycemic control, and HbA1c creates an optimistic outlook on future treatment plans (Whitebird et al., 2017; Morrison et al., 2019; Ventura et al., 2019).

Interpersonal communication and psychological inflexibility were significant predictors of treatment adherence (Akbari et al., 2022; Kılıç et al., 2022). Being able to effectively communicate with persons with diabetes is of the utmost importance. If these individuals better understand their condition and the actions required to improve their HbA1c, they are more likely to engage in the behavioral changes required for more optimal glucose control. Furthermore, psychological flexibility, the opposite of psychological inflexibility, can be defined as a person’s capacity to deal with, accept, and adapt to challenging circumstances (Kılıç et al., 2022). The management of diabetes is nothing short of a challenging circumstance that an individual must adapt and embrace. Therefore, this positive psychology construct may provide another avenue through which diabetes management may be improved.

Furthering the discussion of positive psychology constructs, many studies included in this review linked self-compassion with other tenets of positive psychology, such as mindfulness, self-care, self-efficacy, and family empowerment (Whitebird et al., 2017; Rahmani et al., 2020; Loseby et al., 2022). This combination of multiple positive psychology constructs makes it difficult to infer if self-compassion alone is the reason for the outcomes described in the literature. However, the fact that these constructs can be improved through interventions provides a promising outlook for future research and management plans, as all interventions included in this review reported a meaningful improvement in diabetes management and HbA1c levels following the intervention (Friis et al., 2016; Whitebird et al., 2017; Karami et al., 2018; Rahmani et al., 2020).

Factors identified that might influence self-compassion’s role in diabetes management included gender and diabetes duration (Ringdahl, 2019; Charzyńska et al., 2020). Given that self-compassion is known to be significantly lower in women than in men and that most of the publications included in the review mainly consisted of female populations, caution should be exercised when interpreting the findings of these studies (Yarnell et al., 2018; Ferrari et al., 2022). Ringdahl (2019) identified that gender role conflict may influence any potential relationship identified between self-compassion and diabetes-related health outcomes, of which diabetes management is one. Thus, it is necessary to consider such a factor when investigating the role of self-compassion in managing diabetes. Furthermore, Charzyńska et al. (2020) state that diabetes duration influenced levels of self-coldness, a negative aspect of self-compassion. Most of the publications in this study consisted of participants who had been living with diabetes for a significant period. Given that the longer an individual has diabetes, the more likely they are to understand its causes, course, and treatment, it must be considered that HbA1c levels and glycemic control would not be truly representative of newly diagnosed individuals that are still trying to understand their diabetes and how to manage it effectively. Therefore, it would be beneficial to identify when, in the patient’s diagnosis, self- compassion training would be the most effective; and to identify if an individual’s gender influences how receptive they are to self-compassion interventions and engaging in self-compassionate acts.

Strengths of this review study include the extensive nature of the literature searches, including the most relevant data sources, and using research specialists. Furthermore, many of the studies identified were able to report on changes related to the pre-to-post implementation of their intervention.

The limitations of this study include that 11 of the 16 identified articles employed a cross-sectional design in which causality and lack of a longitudinal follow-up restrict the interpretations that could be made. Additionally, the surplus of quantitative studies could be considered a limitation as the individual’s voice is lost, and we need to get a rich sense of the individual’s perspective and beliefs on the topic under investigation. Another limitation is the range of countries represented in this rapid review (USA, New Zealand, Iran, UK, Australia, and Poland). The results could likely be generalized to other countries; however, more South American, and African-based publications need to be published. Therefore, it is questionable whether these findings would be applicable in a developing context and whether similar outcomes or results would be found among these populations.

Although there appeared to be sufficient literature available, few studies investigated the direct causal relationship between self-compassion and diabetes management and, instead, studied a variety of factors which could influence diabetes management. Therefore, it is recommended that further research is conducted to ascertain if there is a direct causal relationship between self-compassion and diabetes management, as suggested by current research available.

The articles in this review frequently referred to diabetes-related health outcomes or improved psychological outcomes, but few gave information on how these individuals manage their diabetes. These diabetes management behaviors would influence all the variables of interest in these studies, and this focus is missing as only outcomes are reported, not the processes. Therefore, future research must focus on the mechanisms and processes by which these improvements are made.

Of note is the abundance of women in the samples of the publications identified in this review and the use of self-reporting HbA1c levels (Friis et al., 2016; Ferrari et al., 2017; Whitebird et al., 2017; Kane et al., 2018; Karami et al., 2018; Tanenbaum et al., 2018; Ringdahl, 2019; Ventura et al., 2019; Charzyńska et al., 2020; Rahmani et al., 2020). It is vital that future research is conducted in which a more representative sample is used and that more reliable forms of HbA1c data are used, as participants may have over-or under-reported their HbA1c levels to prevent prejudice or stigma.

Lastly, this promising example of a positive psychology perspective, namely self-compassion, in diabetes and its management encourages future research into the role positive psychology may play in managing chronic illnesses such as diabetes.

## Conclusion

This review identified 16 publications in which the relationship between self-compassion and diabetes management was investigated. Self-compassion was found to be associated with improved outcomes in regimen adherence, HbA1c levels, and psychological well-being. However, many psychological variables were identified that mediate this relationship. It was valuable to have identified that self-compassion can be improved through interventions. Multiple publications implemented various programs with the main aim of increasing the participant’s level of self-compassion. The fact that these programs were successful creates a promising outlook for future studies to implement the same approach. Other factors, such as gender and diabetes duration, were also identified as influencing self-compassion among individuals. Further research is needed in which extraneous factors and variables are controlled to ensure that self-compassion does in fact influence an individual’s diabetes management. Based on the publications found in this review, the effect of self-compassion on diabetes management looks promising.

## Author contributions

CS acted as the primary reviewer and ED as the secondary reviewer. CS and ED were involved in the selection and appraisal of the literature. CS conceptualized the study, reviewed the literature, coded the data, and wrote the final research report. ED supervised the rapid review process and acted as the co-coder of the data. All authors contributed to the article and approved the submitted version.

## Conflict of interest

The authors declare that the research was conducted in the absence of any commercial or financial relationships that could be construed as a potential conflict of interest.

## Publisher’s note

All claims expressed in this article are solely those of the authors and do not necessarily represent those of their affiliated organizations, or those of the publisher, the editors and the reviewers. Any product that may be evaluated in this article, or claim that may be made by its manufacturer, is not guaranteed or endorsed by the publisher.
